# Low-Dose Ionizing Radiation and Thyroid Diseases and Functional Modifications in Exposed Workers: A Systematic Review

**DOI:** 10.3390/jcm14020588

**Published:** 2025-01-17

**Authors:** Corrado Colaprico, Francesca Lomartire, Ivana Raccio, Giorgia Mantione, Salvatore Ammirati, Giuseppe La Torre

**Affiliations:** Department of Public Health and Infectious Diseases, Sapienza University of Rome, 00185 Rome, Italy; corrado.colaprico@uniroma1.it (C.C.); lomartire.1847039@studenti.uniroma1.it (F.L.); ivana.raccio@uniroma1.it (I.R.); giorgia.mantione@uniroma1.it (G.M.); salvatore.ammirati@uniroma1.it (S.A.)

**Keywords:** thyroid, low-dose ionizing radiation, workplace, cancer, review

## Abstract

**Background/Objectives:** With technological development, ionizing radiation has found applications in numerous occupations. However, the determination and quantification of the damage resulting from exposure to it remains rather unclear, along with the damage to particular organs. The aim of this systematic review was to investigate the relationship between low-dose ionizing radiation (LDIR) in exposed workers and possible functional changes and cancer development in the thyroid gland. **Methods**: We included observational studies evidencing the correlation under study. Data extraction and analysis was conducted on all included studies. The research strategy included three electronic databases (PubMed, Scopus, and Web of Science). The systematic review followed PRISMA guidelines, and the research protocol was submitted to PROSPERO (CRD:42023425839). **Results**: The search initially yielded 166 articles and, once duplicates and irrelevant articles were removed, a total of 15 useful articles were reviewed. Qualitative analysis of the studies showed that the TSH value does not change following exposure, while a reduction in fT3 and an increase or reduction in fT4 can be observed. Furthermore, the correlation between thyroid cancer and occupational exposure to radiation was not shown with certainty, but there was some evidence of increased gland volume and nodule formation. **Conclusions**: Even at low doses, ionizing radiation adversely affects thyroid activity. In this regard, new studies should be carried out in order to further investigate and define this issue and, consequently, outline useful measures to ensure the protection of workers in contact with this particular physical agent.

## 1. Introduction

In the modern world, ionizing radiation (IR) plays an increasingly prominent role. Humans are constantly exposed to these rays through the environment, their occupation, medical use, or other sources [1]. Sensitivity to radiation varies from organ to organ. The bone marrow and thyroid are those most susceptible to radiation-induced transformation, which is why certain forms of leukemia and thyroid cancer are the neoplasms that occur more frequently and earlier in people exposed to IR [2]. Although the effects of high doses of ionizing radiation (HDIRs) are well known, the effects of low doses of ionizing radiation (LDIRs) on these organs, in particular on the thyroid, still remain ambiguous [3]. In order to unravel these contrasts, a number of studies and reports have been conducted on different populations, starting with the pediatric population [4,5]. In order to provide a large-scale international assessment related to mortality risks from prolonged exposures to low-dose and low-dose-rate ionizing radiation, the International Nuclear Workers Study (INWORKS) was undertaken [6]. The last update of this study shows an increase in the excess relative rate of solid cancer mortality with increasing cumulative exposure to ionizing radiation at the low dose rates typically encountered by French, UK, and US nuclear workers [7]. In light of this, it is clear that people professionally exposed to radiation must be reliably protected continuously from IR: for example, no solid evidence has been provided for an increase in thyroid cancer related to exposure to 131I in cohorts of patients with exposure to it therapeutically and diagnostically [8]. For the protection of workers from exposure to artificial and natural IR, in Italy, the Legislative Decree (D.Lgs.) 101/2020 is in effect, which amended Article 180 paragraph 3 of D.Lgs. 81/08. The decree divides exposed workers into two categories: A (a dose greater than 6 mSv per year up to 20 mSv) and B (workers who may receive a dose of between 1 mSv and 6 mSv per year). Two figures are identified within the decree, namely, the qualified expert, with the role of implementing the principles of radiation protection and physical surveillance, and the occupational physician, responsible for medical surveillance, with the task of taking any post-exposure measures [9]. Since the thyroid is an endocrine organ and highly sensitive to radiation, it is important to assess whether LDIR can cause alterations in its function. The aim of this systematic review, in fact, is to investigate the relationship between exposure to LDIR and possible functional changes and cancer development in the thyroid gland in exposed workers.

## 2. Materials and Methods

A systematic review was conducted to investigate the effects of LDIR exposure on thyroid functionality. This review was recorded in PROSPERO (the International Prospective Register Of Systematic Reviews), and the registration number is CRD42023425839. This study was conducted according to the Preferred Reporting Items for Systematic Reviews (PRISMA) guidelines [10].

### 2.1. Search Strategy

The identification of studies relevant to this review was achieved by searching electronic databases of the published literature, including PubMed, Scopus, and Web of Science. The keywords used in the electronic databases were “thyroid AND low AND dose AND ionizing AND radiation AND (function OR cancer)”. The search was undertaken with no language or date of publication restrictions. The article search and data extraction were carried out during the period between 15 April 2023 and 31 May 2023.

### 2.2. Study Selection

The review process was carried out using a multi-stage approach. Five authors independently conducted duplicate screening and removal and, as computer software, used ZOTERO. Then, after title and abstract screening, full-text articles were assessed to determine whether they met the inclusion criteria. If an included publication was not available as the full text in English, the corresponding author was contacted to verify whether the eligibility criteria were met.

### 2.3. Inclusion and Exclusion Criteria

The inclusion criteria were as follows: (1) studies involving workers exposed to LDIR; (2) the focus of the research was the effect of LDIR on thyroid function and cancer development. The exclusion criteria were as follows: (1) irrelevance to the research topic; (2) studies involving workers not exposed to LDIR; and (3) articles studying the effect of LDIR on other organs.

### 2.4. Data Extraction

Data extraction was conducted independently by five reviewers, extracting data from all included studies. In order to confirm the relevance of the studies and, consequently, extract their characteristics, the authors developed a data collection form in which the following information was included: name of the first author, title, country, year of publication, study design (cohort, case–control, cross-sectional, RCT), type of workplace, sample size (total), sample size (with problem), type of exposure (unit of measurement used, exposure intervals…), type of thyroid problem (cancer, nodules, hormone variations), main results, and assessment of the quality of the study. In cohort studies, the measure of association considered was the relative risk (RR), while in case–control studies, it was the odds ratio (OR). To ensure accurate data collection, the data extracted were compared independently by each reviewer. Discrepancies and disagreements were discussed and resolved through a consensus session with a third-party researcher.

### 2.5. Quality Assessment

A quality assessment of the observational studies was carried out using the Newcastle–Ottawa Scale (NOS). This is a validated, easy-to-use scale of 8 items in three domains, selection, comparability, and exposure/outcome, for case–control or cohort studies. Each item can be given one point, except comparability which has the potential to score up to two points. Studies are rated from 0 to 9, with studies rated 0–3 being poor-quality, 4–6 being fair-quality, and 7–9 being good/high-quality.

A Spearman correlation coefficient was calculated considering the association between quality assessment scores and the year of publication and sample size of the studies. Finally, a weighted (by sample size) regression analysis was carried out using the quality as the dependent variable and the year of publication as the independent variable. The result is described using the β coefficient (p).

## 3. Results

### 3.1. Search Results Summary

**Figure 1:**
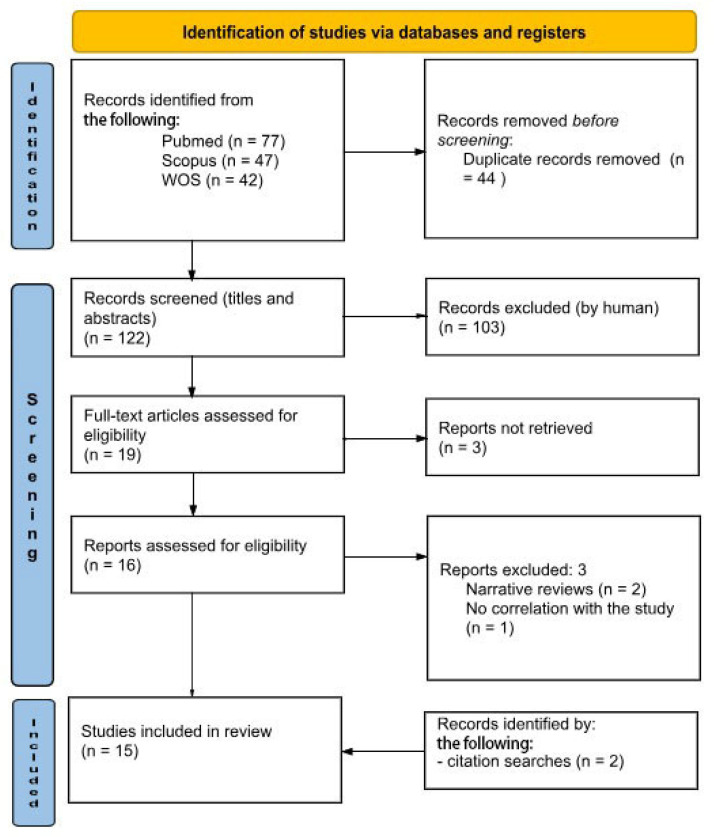
PRISMA 2020 flow diagram.

Research began in April 2023. The initial search across different electronic databases yielded 166 citations. First, a total of 44 duplicate papers were excluded, accompanied by the removal of 103 publications in the title/abstract screening. Among the nineteen full-text articles screened, three were not included. Finally, among the sixteen articles selected and evaluated for eligibility, three reports, upon further reading of the text, were excluded for the following reasons: two were narrative reviews, and the third had no useful correlation with the aim of our study. Through a search of literary citations, a further two useful articles were identified for review. At the end of the process, 15 studies remained for qualitative analysis (Figure 1).

### 3.2. Characteristics of Included Studies

Fifteen studies were selected for our systematic review (Table 1). Their publication dates ranged from 1997 to 2022. In terms of study design, six were cross-sectional, and nine were cohort studies, with 1,040,763 people included in our review. The total worker population in the cohort studies was 1,038,545 people, while in case–control studies, 2218 patients were considered. In particular, for case–control studies, hospital controls were used in the studies of Vázquez Rivas [11], El-Benhawy [12], and Cioffi [13], and community controls were used in the studies of Adibi [14], Gudzenko [15]. The studies were conducted mainly in Europe with seven studies, then four in Asia, three in the USA, and one in Africa. The workplaces considered by the studies were as follows: hospitals and healthcare with ten studies, nuclear power plants with six studies, universities and academia with two studies, industrial services with three studies, the war industry and barracks with three studies, and public services (which included drivers, workers in airports, stations, etc.) with one study. The thyroid disorders considered consisted of thyroid cancer, thyroid nodules, and changes in thyroid hormones (TSH, fT3, fT4). While in five studies [11,13,15,16,17,18,19] workers were divided into groups according to the exposure intervals they were subjected to, for the remaining studies, we have no clear data.

### 3.3. Quality Assessment

The quality of each study was evaluated independently by two reviewers using the NOS scale: for case–control studies, the lowest rating was 7, the highest 8; for cohort studies, the lowest rating was 5, the highest 7. The average rating for all studies was 6.73.

**Figure 2:**
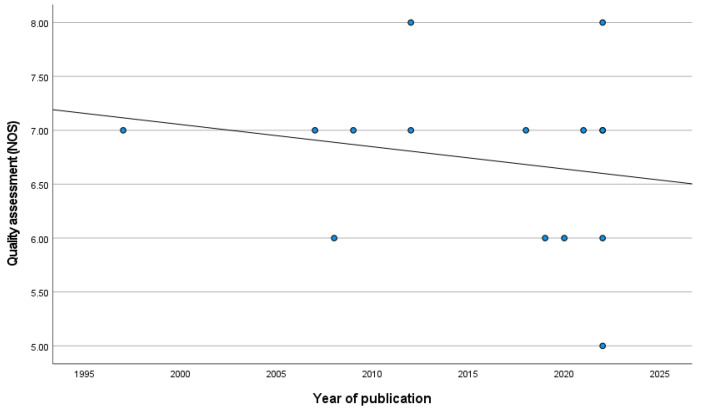
Correlation between year of publication and methodological quality of the studies.

The case–control studies (six) had a mean value of 7.17 (SD = 0.75), while the cohort studies (nine) had a mean value of 6.44 (SD = 0.73) (*p* = 0.210). Details of the ratings using the NOS scale can be found in Supplementary Materials S1. Interestingly, the year of publication is inversely correlated with the methodological quality of the studies, even if this is not statistically significant (r = −0.197; *p* = 0.481) (Figure 2). However, this correlation has a positive trend when the analysis is weighted by the sample size (β = 0.055; *p* < 0.001).

**Figure 3:**
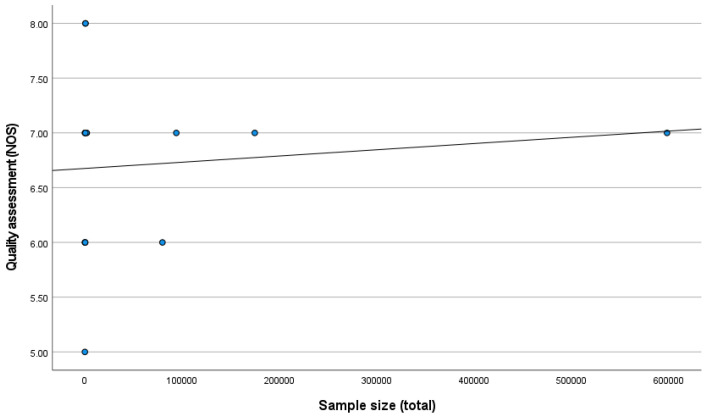
Correlation between sample size and methodological quality of the studies.

A positive correlation was found between the sample size and quality assessment (r = 0.111; *p* = 0.705) (Figure 3).

### 3.4. Thyroid Cancer

Seven studies [15,16,18,19,20,23,24] evaluated the possible association between LDIR and thyroid cancer. In the first study, thyroid cancer was statistically significant in women working with radiation in medical and research institutions and in industry and in all nuclear energy workers, and there was a significant association with dose (ERR = 20.4 for Sv, 90% CI = 8–60, one-tailed *p* = 0.049). In the second study of nine healthcare workers with papillary thyroid carcinoma (PTC) exposed to LDIR, BRAF V600 genetic alterations were revealed in four cases, including one with RET/PTC1 fusion, and in one case, point mutations and fusions were found. In the third study, the risk of thyroid cancer was found to be increased for men and women compared with the general population: in particular, there appears to be an inversely proportional correlation according to cumulative badge dose categories (half of all cases have thyroid cancer for doses <1 mSv). In the fourth study, thyroid cancer risk was not associated with the cumulative, occupational IR dose; restricting to papillary thyroid cancer yielded nearly identical results. In the last study, a non-statistically significant SMR was found for thyroid cancer; however, there is weak evidence of thyroid cancer in two cases with a cumulative dose above 400 mSv. In the last two studies, a significant dose–response relationship was observed between the radiation dose to the thyroid among the highest dose categories, but there was no evidence about LDIR and thyroid cancer.

### 3.5. Thyroid Nodules

Three studies [12,18,19] evaluate the association between thyroid nodules and LDIR exposure. In the first study, a significant association (*p* = 0.005) was reported between the presence of thyroid nodules in the exposed group compared with the control group, and in the second study, the prevalence of thyroid nodules in cases and controls was 22.6% and 24.6%, respectively (*p* > 0.05). It is noteworthy that both studies involved employees of healthcare facilities. In the last study, on the other hand, conducted on workers at the Chernobyl nuclear power plant following the nuclear accident, the presence of nodules was not significantly associated with the documented radiation dose from external sources.

### 3.6. Thyroid Hormones

There are five studies that have evaluated the association between hormonal changes and LDIR exposure [11,12,13,21,22]. In the first study, a significant increase in fT3 compared to the controls and anti-TPO is shown in subjects exposed to 131I; also reported was an increase in the mean thyroid volume in the exposed group. In the second study, there was no significant difference in hormonal changes between the exposed and unexposed groups, although higher levels of TSH were found in the category B and A exposed groups compared with the TSH levels in the control group; on the other hand, a statistically significant difference was found in the five-year dose of the personal dosimeters between the medium/high- and low-risk categories, being 0.1 ± 0.3 in the low-exposure zone and 0.9 ± 1.4 in the medium/high-exposure zone. In the third study, exposed workers presented significantly higher levels of TSH and significantly lower levels of fT3 and fT4 than non-exposed workers. In the fourth study, the data showed no variation in TSH levels related to occupational exposure between T1 and T2, an increase in fT3 hormone values, and a decrease in fT4. In the latest study, the authors observed declines in fT3 and fT4 over the study period but not in TSH: they also observed an increase in the TSH level after the ninth year of follow-up.

Finally, the study by Cardis [25] does not consider a specific thyroid-related problem but reports the deaths caused by thyroid problems of workers who were exposed to LDIR: all workers who died of thyroid problems were exposed to doses below 50 mSv. The Standardized Mortality Ratio (SMR) appeared to be maximum for a cumulative dose of 10 mSv (RR at 100 mSv: 0,91 [0.12, 2.84]).

## 4. Discussion

The purpose of this systematic review was to investigate the effects of LDIR exposure on thyroid functionality and cancer development in exposed workers. The health effect of LDIR has always been a source of heated debate. Although unequivocal evidence of the carcinogenic power of IR has been achieved, so much so that the IARC [26] has for many years placed it in class 1 of “carcinogenic agents for humans”, the results of the epidemiological studies conducted to date reveal a more complex, and in some areas controversial, picture.

And our review reflects this trend. We first looked for articles evaluating the possible association between LDIR and thyroid cancer, with mixed results. Two studies, both conducted in Korea, report a significant association between LDIR and cancer, while the other studies report no significant association. The study conducted by Duque also reports the presence of genetic alterations in some patients with papillary thyroid carcinoma. One part of the literature reports, in general, that the primary ill-health caused by low to moderate doses of IR is cancer, although the possibility of non-cancer effects (particularly cardiovascular disease) is of increasing concern [27]. In contrast, as far as thyroid cancer is concerned, it is still necessary to establish whether LDIR causes damage to the organ, as it is particularly radiosensitive, which is why further research on this issue is needed.

Regarding the correlation between the presence of nodules and exposure to LDIR, the literature provides few significant data; in particular, only two studies show an association between exposure and LDIR in a healthcare setting. Again, there are numerous studies in the literature demonstrating an association between HDIR and thyroid nodules [28,29], but very little has been analyzed in relation to the association with LDIR.

Finally, regarding the association between LDIR and thyroid hormones, the current literature about the fT3 trend is very conflicting, as it increased in two studies and decreased in another two, and it is concordant in the fT4 trend, as it increased in three studies, and the TSH trend, as it increased in three studies and remained unchanged in one. A recent study conducted in China on a large sample of healthcare workers has clarified the relationship between LDIR and the thyroid gland; in particular, it was seen that long-term exposure to LDIR had an effect on thyroid abnormalities in medical radiation workers. Among them, females, physicians, and those working in the department of diagnostic radiology were at a higher risk of abnormal thyroid function; being female, having increased years of radiation work, and radiation exposure onset at age ≥ 30 years were associated with a higher risk of reporting abnormal thyroid morphology [30].

Ours is the first review analyzing the association between LDIR exposure and thyroid function. Among the strengths, we mention the large sample size and cohort design of most of the studies, some of which were based on data obtained from official national registers. Furthermore, numerous aspects relating to the thyroid (cancer, nodules, hormones) were taken into consideration in order to have the most complete view possible regarding the correlation with LDIR. Another strength of our study is the quality of the studies considered, which is moderate. However, there are limitations. First of all, there are no studies from Oceania and South America, as they are not present in the literature. The settings analyzed by the studies are very different and vary from the controlled and standardized hospital environment to nuclear power plants and industries in which the ways of collecting worker data could be different. Moreover, there were no data regarding previously treated thyroid diseases. Finally, all included studies were cross-sectional or cohort studies, so although it is possible to find associations, it is not possible to establish what the underlying pathophysiological mechanisms are.

## 5. Conclusions

Studies present different results regarding the correlation between exposure to LDIR and thyroid dysfunction. From our review, it emerges that in the literature there are few studies relating to the correlation between LDIR and thyroid function, and these studies present data that are sometimes conflicting with each other. It is clear, however, that even at low doses, it negatively affects the activities of the thyroid. In this regard, it is necessary to collect more documentation, carry out new studies in order to delve deeper and define this topic, and, consequently, outline useful measures with the aim of ensuring the protection of workers in contact with this particular physical agent.

## Figures and Tables

**Table 1:** Characteristics of included studies.

1st Author	Country	Study Design	Type of Workplace	Sample Size	Thyroid Cancer	Thyroid Nodules	TSH, fT3, fT4 Variations	Main Results	Quality Assessment (NOS)
Ahn Y, 2008 [16]	South Korea	Cohort	Universities and academia, hospitals and healthcare, nuclear power plants, industrial services, war industry and barracks, and public services	79,679	X	-	-	Thyroid cancer was statistically significantly elevated in female radiation workers in medical (SRR = 2.90, 95% CI = 1.05–7.94) and research institutions (SRR = 3.91, 95% CI = 1.36–11.0) and industry (SRR = 5.07, 95% CI = 1.56–15.6) and in all nuclear power workers (SRR = 2.59, 95% CI = 1.33–5.13), and there was a significant association with dose (ERR = 20.4 per Sv, 90% CI = 8 to 60, one-tailed *p* = 0.049).	6
El-Benhawy SA, 2022 [12]	Egypt	Case–control	Hospitals and healthcare	120	-	X	X	Participants from the group exposed to radioiodine 131I had significantly higher fT3 mean values than participants from the group of non-exposed healthy professionals (3.01 ± 0.41 vs. 2.76 ± 0.38, *p* = 0.047 *). Regarding anti-TPO, their mean values were substantially higher in participants from group I than in participants from group III (35.61 ± 82.35 vs. 8.40 ± 1.26, *p* < 0.001 *, respectively). The mean thyroid volume (ml) was significantly larger in participants from group I, in comparison to participants from group III (10.32 ± 3.42 vs. 4.62 ± 1.13, *p* < 0.001 *). The thyroid nodule percentage in group I was significantly higher than in the control group (*p* = 0.005 *).	7
Vazquez Rivas F, 2022 [11]	Spain	Case–control	Hospitals and healthcare	186	-	-	X	No statistically significant relationship was found between the levels of thyroid hormones and the occupational exposure to radiation in PER A and PER B, though higher levels of TSH were found in the exposed groups PER B (2.6 ± 1.4) and PER A (2.7 ± 1.3) compared to the TSH levels in the control group (2.4 ± 1.5).	7
Cioffi DL, 2020 [13]	Italy	Case–control	Hospitals and healthcare	180	-	-	X	Exposed workers presented significantly higher levels of TSH **(β = 0.88, %95CI [0.43, 1.33], *p* = 0.001)** and significantly lower levels of fT3 (β = −0.61, %95CI [−0.74, −0.47], *p* < 0.001) and fT4 (β = −0.10, %95CI [−0.16, −0.04], *p* = 0.001) than non-exposed workers.	6
Duque C S, 2022 [20]	USA	Cohort	Hospitals and healthcare	9	X	-	-	Nine healthcare workers with papillary thyroid carcinoma exposed to LDIR. The molecular analysis of surgical samples of PTCs was informative and revealed genetic alterations in five patients. BRAF V600E was found in four (67%) cases, whereas RET/PTC1 fusion was found in one (17%), and one sample (17%) was wild-type for point mutations and fusions.	5
Sernia S, 2022 [21]	Italy	Cohort	Universities and academia and hospitals and healthcare	121	-	-	X	Data showed no variation in TSH levels related to occupational exposure, an increase in fT3 hormone values in HCW and residents, and a decrease in fT4 in HCW (these results are in conflict with previous studies, in which both free hormones decreased with a concomitant increase in TSH).	6
Wong YS, 2019 [22]	Taiwan	Cohort	Hospitals and healthcare	326	-	-	X	We observed declines in fT3 and fT4 over the study period but not in TSH. In addition, we found negative dose–response relationships between exposure duration and declines in the serum levels of fT3 (a change of −0.037 ng/mL/year after adjusting for sex and age at the beginning of follow-up; 95%CI = −0.042, −0.032 ng/mL/year) and fT4 (−0.115 μg/dL/year; 95%CI = −0.140, −0.091 μg/dL/year). We also observed an increase in the TSH level (0.683 μIU/mL/year; 95% CI = 0.151, 1.214 μIU/mL/year) after the ninth year of follow-up.	6
Adibi A, 2012 [14]	Iran	Case–control	Hospitals and healthcare	595	-	X	-	The prevalence of thyroid nodules in the case and control groups was 22.6% and 24.6%, respectively (*p* > 0.05). Although thyroid nodules were significantly more prevalent in females in the control group, no such difference was observed between females and males in the case group (*p* > 0.05). The number of thyroid nodules (single or multiple) and calcifications was not different between the two groups (*p* > 0.05). In addition, the hypoechogenicity of thyroid nodules was not different between the two groups (*p* > 0.05).	8
Inskip PD, 1997 [17]	USA	Cohort	Nuclear power plants	1984	-	X	-	The presence of nodules was not significantly associated with the documented radiation dose from external sources [p(1) = 0.70; Excess RR (ERR) = −0.01/cGy; 95% CI: −0.02, 0.01]. Among men with nodules, the mean dose was 10.2 cGy, and for those without nodules, the mean was 10.9 cGy. The mean for the total population was 10.8 cGy, and the maximum recorded dose for any worker was 61 cGy.	7
Lee WJ, 2021 [23]	South Korea	Cohort	Hospitals and healthcare	93,920	X	-	-	The risk of thyroid cancer from workers’ exposure to low-dose ionizing radiation appears to be increased for men and women compared with the general population. In particular, there appears to be an inversely proportional correlation according to cumulative badge dose categories (half of all cases have thyroid cancer for doses <1 mSv).	7
Kitahara CM, 2018 [24]	USA	Cohort	Hospitals and healthcare	89,897	X	-	-	Thyroid cancer risk was not associated with cumulative, occupational ionizing radiation dose to the thyroid gland in fully adjusted models (ERR/100 mGy = −0.05, 95% CI < −0.10, 0.34). Restricting to papillary thyroid cancer (303 cases) yielded nearly identical results (ERR/100 mGy = −0.05, 95% CI < 0.10, 0.39).	7
Muirhead CR, 2009 [18]	United Kingdom	Cohort	Nuclear power plants, industrial services, and war industry and barracks	174,541	X	-	-	Both NRRW−2 and NRRW-3 found a non-statistically significantly raised SMR for thyroid cancer but no association between mortality and dose. As thyroid cancers are usually not fatal, the incidence data should be more informative. Although there was weak evidence of a trend with external dose in thyroid cancer incidence (one-sided P ¼ 0.079), this was driven primarily by two cases with a cumulative dose above 400 mSv.	7
Cardis S, 2007 [25]	France	Cohort	Nuclear power plants, industrial services, and war industry and barracks	598,068	-	-	-	All workers who died of thyroid problems were exposed to doses below 50. The Standardized Mortality Ratio (SMR) appears to be maximum for a cumulative dose of 10 mSv (RR at 100 mSv: 0.91 (0.12, 2.84)).	7
Gudzenko N, 2022 [15]	Ukraine	Case–control	Nuclear power plants	607	x	-	-	Total thyroid dose was associated with nonsignificantly increased risk of thyroid cancer, with some evidence that the magnitude of radiation risk varied with time since exposure. Although category-specific risk estimates were not significant, the OR was highest among the highest dose categories. There was also a nonsignificant elevation in risk for FTC based on a small number of cases.	8
Kesminiene A, 2012 [19]	France	Case–control	Nuclear power plants	530	x	-	-	Most subjects received low doses (median 69 mGy). A statistically significant dose–response relationship was found with total thyroid dose. The Excess Relative Risk (ERR) per 100 mGy was 0.38 [95% confidence interval (CI): 0.10, 1.09]. A significant dose–response relationship was observed between radiation dose to the thyroid received in adulthood and the risk of subsequent thyroid cancer, with a significantly increased risk at doses of 300 mGy or above.	7

## Supplementary Materials

The following supporting information can be downloaded at: https://www.mdpi.com/article/10.3390/jcm14020588/s1.
