# Supplementary material for: Low-Dose Ionizing Radiation and Thyroid Diseases and Functional Modifications in Exposed Workers: A Systematic Review
Source: J Clin Med. 2025 Jan 17;14(2):588. doi: 10.3390/jcm14020588 (PMC11766329; doi:10.3390/jcm14020588)
Supplement: Supplementary file 1 [file jcm-14-00588-s001.zip › jcm-3329099-supplementary.pdf]

Supplementary Materials S1 – Quality Assessment NOS

Cohort studies

**1st Author**	**Year of publication**	NOS S1	NOS S2	NOS S3	NOS S4	NOS C1	NOS O1	NOS O2	NOS O3	TOTAL
Ahn Y	2008	1	0	1	1	1	1	1	0	6
Duque C S	2022	1	0	1	0	1	1	1	0	5
Sernia S	2022	1	1	1	1	1	1	0	0	6
Wong YS	2019	1	1	1	0	1	1	1	0	6
Inskip P D	1997	1	1	1	0	1	1	1	0	6
Lee WJ	2021	1	1	1	1	1	1	1	0	7
Kitahara CM	2018	1	1	1	1	1	1	1	0	7
Muirhead CR	2009	1	1	1	1	1	1	1	0	7
Cardis S	2007	1	1	1	1	1	1	1	0	7

Case-control studies

**1st Author**	**Year of publication**	NOS S1	NOS S2	NOS S3	NOS S4	NOS C1	NOS E1	NOS E2	NOS E3	TOTAL
Vázquez Rivas F	2022	1	1	1	1	1	1	1	0	7
El-Benhawy SA	2022	1	1	1	1	1	1	1	0	7
Cioffi DL	2020	1	1	1	1	1	1	1	0	7
Adibi A	2012	1	1	1	1	1	1	1	1	8
Gudzenko N	2022	1	1	1	1	1	1	1	1	8
Kesminiene A	2012	1	1	1	1	1	1	1	0	7
